# Prevalence of Newcastle Disease Virus in Commercial and Backyard Poultry in Haryana, India

**DOI:** 10.3389/fvets.2021.725232

**Published:** 2021-11-04

**Authors:** Vinay G. Joshi, Deepika Chaudhary, Nitish Bansal, Renu Singh, Sushila Maan, Nand K. Mahajan, Chintu Ravishankar, Niranjana Sahoo, Sunil K. Mor, Jessica Radzio-Basu, Catherine M. Herzog, Vivek Kapur, Parveen Goel, Naresh Jindal, Sagar M. Goyal

**Affiliations:** ^1^Department of Animal Biotechnology, Lala Lajpat Rai University of Veterinary and Animal Sciences, Hisar, India; ^2^Department of Veterinary Public Health & Epidemiology, Lala Lajpat Rai University of Veterinary and Animal Sciences, Hisar, India; ^3^Department of Veterinary Microbiology, Kerala Veterinary and Animal Sciences University, Pookode, India; ^4^College of Veterinary Science and Animal Husbandry, Odisha University of Agriculture and Technology, Bhubaneswar, India; ^5^Department of Veterinary Population Medicine and Veterinary Diagnostic Laboratory, University of Minnesota, St. Paul, MN, United States; ^6^The Huck Institute of the Life Sciences, University Park, PA, United States; ^7^Department of Animal Science, The Pennsylvania State University, University Park, PA, United States; ^8^Directorate of Research, Lala Lajpat Rai University of Veterinary and Animal Sciences, Hisar, India

**Keywords:** Newcastle disease virus, surveillance, commercial poultry, backyard birds, RT-PCR

## Abstract

Newcastle disease virus (NDV) causes Newcastle disease (ND) in poultry. The ND is a highly contagious disease, which is endemic in several countries despite regular vaccination with live or killed vaccines. Studies on NDV in India are mostly targeted toward its detection and characterization from disease outbreaks. A surveillance study was undertaken to determine NDV prevalence throughout the state of Haryana from March 2018 to March 2020 using a stratified sampling scheme. The state was divided into three different zones and a total of 4,001 choanal swab samples were collected from backyard poultry, commercial broilers, and layers. These samples were tested for the M gene of NDV using real-time RT-PCR. Of the 4,001 samples tested, 392 were positive (9.8% apparent prevalence; 95% CI: 8.9–10.8%) for the M gene. Of these 392 M gene positive samples, 35 (8.9%; 95% CI: 6.4–12.3%) were found to be positive based on F gene real-time RT-PCR. Circulation of NDV in commercial and backyard poultry highlights the importance of surveillance studies even in apparently healthy flocks. The information generated in this study should contribute to better understanding of NDV epidemiology in India and may help formulate appropriate disease control strategies for commercial and backyard birds.

## Introduction

Newcastle disease (ND) is a highly contagious disease of domestic and wild birds worldwide (1). The disease is caused by the Newcastle disease virus (NDV), also known as avian paramyxovirus-1 (AMPV-1), under the genus *Avian orthoavulavirus*-1 (2). It is a non-segmented, negative-sense, single-stranded RNA virus with average genome sizes of 15,186–15,198 nucleotides (3). The virulence of NDV depends upon the amino acid sequence at the cleavage site of the fusion protein (F), which plays a vital role in virus entry and pathogenesis (4). On this basis, the NDV strains are divided into highly virulent velogenic, moderately virulent mesogenic, and avirulent lentogenic. The consensus amino acid sequence of the F protein cleavage site of velogenic and mesogenic strains is 112R/K-R-Q-R/KRY/F117; whereas that of lentogenic strains is 112G/E-K/R-Q-G/ERY/L117. Due to these special characteristic variations observed in the circulating NDV strains, demonstrating such genotypic variation in the fusion gene (F gene) is a preferred method for NDV diagnosis and virus typing.

At present, class II NDV is classified into 18 genotypes and multiple sub-genotypes (5). Due to vast variations in circulating NDV, outbreaks of ND are common in different species of birds (6) and the disease is considered a major limiting factor for poultry production in low and middle-income countries (LMIC). The economic burden is due to high mortality and morbidity including the cost of vaccination and implementation of biosecurity measures (6). The major thrust for NDV diagnosis is to detect virulent viruses rapidly in an effort to control outbreaks. This strategy allows for identification of predominantly circulating genotypes of NDV in a particular region.

Outbreaks of ND in commercial poultry and backyard birds have been reported from many parts of India (7–10). Vaccines are used regularly in commercial poultry to reduce the devastating consequences of such outbreaks. Unfortunately, the billions of doses used for vaccination are likely to release live virus into the environment putting pressure on virus evolution (11). In the absence of clear data on NDV surveillance and clustering of NDV outbreak patterns in poultry, it becomes difficult to implement preventive and control measures to contain frequent outbreaks of ND. Surveillance data are also important to understand the persistence, transmission, and evolution of NDV, which can help inform relative risk assessment and the design and implementation of long-term sustainable prophylactic strategies.

For large-scale NDV surveillance studies, a molecular approach is needed. Many studies have suggested the use of conserved real-time RT-PCR (rRT-PCR) probe for initial screening of NDV in the population (12, 13). Real-time RT-PCR based on the conserved matrix gene (M gene) probe is universally used for the detection of NDV in surveillance studies (13). The F gene probe, on the other hand, is used for the differentiation of virulent and avirulent NDV (14, 15). This study reports targeted molecular surveillance of NDV in commercial and backyard poultry in Haryana, India. The objective of this study was to use the surveillance data to estimate risk factors associated with NDV in commercial and backyard poultry.

## Materials and Methods

### Sample Source

Haryana, a northern state of India, is located between 27° 37' to 30° 35' latitude and 74° 28' to 77° 36' longitude. For this study, the state was divided into three different zones (Zones 1–3) (Supplementary Table 1). The approximate population of domestic poultry in zones 1, 2, and 3 is 841, 1,856, and 742 birds per square kilometer, respectively. Stratified sampling was done based on a power of 0.8, a significance level of 0.05, and an effect size of 0.2. The sampling from March 2018 to March 2020 was conducted among apparently healthy commercial and backyard poultry as well as from flocks in which birds exhibited mild respiratory signs or were sick.

### Sample Collection

Choanal swabs from birds were obtained using Dacron swabs, which were then placed in 2.2 mL of sterile Brain Heart Infusion (BHI) broth contained in a sterile pre-labeled 4.5 mL cryovial. The cryovials were placed on ice and transported to the laboratory within 4–24 h of sample collection where they were stored at −80°C until tested. Tissue samples (pool of lungs, trachea, and air sac) from a few dead birds were also collected.

A questionnaire was used at the time of sampling to collect risk factor data for NDV infection (Supplementary Table 2). Relevant variables such as vaccination, flock health, bird health at the time of sampling, housing type, bird type, geographical coordinates, and other details were entered in EpiCollect 5, a free data-gathering software. Bird management was categorized into three different types, namely extensive (<20 backyard birds), semi-intensive (20–200 backyard birds), and intensive (flocks of commercial or backyard poultry with more than 200 birds). The categorization used in this study for backyard poultry (extensive) represents both small extensive scavenging and extensive scavenging poultry production systems as per FAO poultry production classification. The semi-intensive and intensive categorization of backyard birds corresponds to the semi-intensive and small-scale intensive poultry production system of FAO classification, respectively (16).

A total of 4,001 samples were collected [backyard poultry (*n* = 1,562), commercial broilers (*n* = 1,657), and commercial layers (*n* = 782)]. The samples from backyard poultry were collected from 202 family-owned farms in 74 villages situated in 15 different districts of the state. Samples from commercial broilers were collected from 172 flocks in 112 villages in 16 districts. Samples from layers were collected from 31 flocks in 16 villages in 8 districts.

### RNA Extraction and Real-Time RT-PCR

For RNA extraction, Mag MAX™ AI/ND Viral RNA extraction kit (Applied Biosystems, USA) was used. This kit is designed for high throughput purification of total RNA from oropharyngeal/tracheal swab samples as well as cultured cells. Qiagen OneStep RT-PCR kit was used for rRT-PCR. All samples were screened for the M gene of NDV using USDA APMV-1 detection primers and probe (Table 1) (13). The efficiency of the M gene real-time probe was determined by testing serial 10-fold dilutions of RNA extracted from a vaccine strain (LaSota) of NDV with known egg infectious dose (EID_50_)_._ The reaction conditions for M gene rRT-PCR were reverse transcription at 50°C for 25 min followed by initial PCR activation for 10 min at 95°C, denaturation at 95°C for 10 sec, and annealing and extension at 60°C for 30 sec. Samples with a cycle threshold (Ct) value of up to and including 35 were considered positive for NDV; all others were considered negative.

**Table 1:** Primers and probes used for M and F gene real-time RT-PCR.

**Specificity and target gene (reference)**	**Oligo name**	**Primer/probe**	**Sequence**
APMV-12003 M gene	M+4100	5' Primer	5'-AGTGATGTGCTCGGACCTTC-3'
(13)	M+4169	Probe	5'-(FAM)TTCTCTAGCAGTGGGACAGCCTGC-(BHQ]-3'
	M-4220	3' Primer	5'-CCTGAGGAGAGGCATTTGCTA-3'
vNDV2003 F gene	VF1	5' Primer	5'GAYTCYATCCGYAGGATACAAGRG 3'
(15)	V probe 1	Probe	5'-(FAM)AARCGTYTCTGYCTCC MGB NFQ 3'
	VR2	3' Primer	5'AACCCCAAGAGCTACACYRCC 3'

Samples positive for the M gene were further tested with the F gene probe (Table 1) to differentiate between virulent and avirulent strains of NDV (15). The F gene probe detects the most prevalent velogenic and mesogenic strains of NDV and can identify five cleavage site motifs (RRQKRF, RRQRRF, RRRKRF, KRQKRF, GRQKRF), targeting important circulating mesogenic/velogenic strains of NDV (15). F gene testing was also done by rRT-PCR using the Qiagen One-Step RT-PCR kit. The reaction conditions were the same as used for M gene amplification. The reaction mixture (12.5 μl) contained 2.5 μl 5x reaction buffer, 0.4 μl forward primer (10 pm), 0.4 μl reverse primer (10 pm), 0.3 μl probe (5 pm), 0.4 μl dNTPs (10 Mm), 0.5 μl enzyme mix, 5 μl nuclease-free water (NFW), and 3 μl template. Samples with a Ct value of <40 were considered positive, Ct values 40 and above were considered negative (15).

Before testing of field samples, the sensitivity of the primer and probe was tested with RNA extracted from LaSota and R_2_B vaccine strains and an archived velogenic NDV isolate. For specificity, RNA from an unrelated virus Infectious bronchitis virus, tested. After ascertaining the suitability of the M gene probe for the detection of lentogenic, mesogenic, and velogenic NDV, sensitivity of the M probe was determined by 10-fold serial dilutions of RNA from LaSota strain; the probe was able to detect 10^1.9^ EID_50_ of the virus. The F gene probe specificity was tested with R_2_B vaccine strain and a genotype VII virus isolate. The lentogenic LaSota strain was used as a negative control. The sensitivity of the F gene assay was found to be 10^2.5^ EID_50_ virus.

### Statistical Analysis

NDV prevalence overall and stratified by risk factors, was calculated along with corresponding 95% confidence intervals (CI) using R statistical software, prop.test function (17). A series of logistic regression models were run using the glm() function in R. Before modeling, 25 rows were removed by filtering out individuals that had housing = “free range” as this was not a dominant housing type of interest. The modeling dataset included 3,976 birds. The outcome was a positive M gene test for a bird and the independent variables of risk factors were selected from the questionnaire data based on expert opinion. Collinearity in the model was checked using the vif() function. The final model included vaccination status, season, and bird type as independent variables with the non-vaccinated, non-migratory season, and commercial layers as the reference levels. The log odds ratio output from the glm() was converted into a relative risk score and accompanying 95% confidence interval using the function odds_to_rr() from the sjstats package (18). This function uses the equation: RR < − OR/[1 − P0 + (P0 ^*^ OR)] from (19–21).

## Results

### M Gene rRT-PCR

By M gene-based rRT-PCR, 392 of 4,001 samples (9.8%; 95% CI: 8.9–10.8%) were found positive for NDV. Of the 4,001 samples collected, 3,536 (88.4%; 95% CI: 87.3–89.3%) belonged to healthy flocks (both commercial and backyard) while 465 (11.6%; 95% CI:10.7–12.7%) were from flocks having sick birds (*n* = 33) and birds with mild respiratory signs (*n* = 432; Table 2). The prevalence of NDV based on M gene detection was higher in flocks with mild respiratory problems (15.7%; 95% CI: 12.6–19.5%) followed by healthy (9.1%; 95% CI: 8.2–10.2%) and sick (3.0%; 95% CI: 0.5–15.4%; Tables 2, 3) flocks. The analysis of data based on bird health revealed that sick birds had a higher prevalence (15.2%; 95% CI: 12.2–18.9%) as compared to the dead (9.6%; 95% CI: 6.6–13.9%) and healthy birds (9.1%; 95% CI: 8.2–10.2%).

**Table 2:** Prevalence of Newcastle disease virus according to flock health status on the basis of M gene detection.

**Flock status**	**No. tested**	**No. positive**	**Per cent**
			**positive**
Sick birds	33	1	3.0
Birds with mild respiratory signs	432	68	15.7
Apparently healthy birds	3,536	323	9.1
Total	4,001	392	9.8

**Table 3:** Newcastle disease virus prevalence among domestic poultry in Haryana.

**Variable**	**Condition**	**Number positive/Number tested**	**Apparent prevalence**	**95% CI**	True prevalence^*^	**95% CI**
Flock status	Healthy	323/3,536	9.13	8.23–10.13	4.86	3.8–6.03
	Sick	1/33	3.03	0.5–15.32	−2.32	−5.7–12.14
	Respiratory	68/432	15.74	12.61–19.48	12.64	8.95–17.03
Bird status	Healthy	302/3,317	9.1	8.17–10.13	4.83	3.73–6.04
	Dead	24/250	9.6	6.54–13.89	5.41	1.81–10.45
	Sick	66/434	15.21	12.14–18.89	12.01	8.39–16.34
Housing	Intensive	277/2,582	10.73	9.59–11.98	6.74	5.4–8.21
	Semi-intensive	63/764	8.25	6.5–10.41	3.82	1.76–6.37
	Extensive backyard	52/655	7.94	6.11–10.26	3.46	1.3–6.19
Zone	1	179/1,356	13.2	11.5–15.11	9.65	7.65–11.89
	2	75/1,350	5.56	4.46–6.91	0.65	−0.64–2.55
	3	138/1,295	10.66	9.09–12.45	6.65	4.81–8.77
Bird type	Backyard	119/1,562	7.62	6.4–9.04	3.08	1.65–4.75
	Commercial broiler	196/1,657	11.83	10.36–13.47	8.03	6.31–9.97
	Commercial layer	77/782	9.85	7.95–12.14	5.7	3.47–8.39

* *Considering diagnostic sensitivity 90% and diagnostic specificity 95%*.

**Figure 1:**
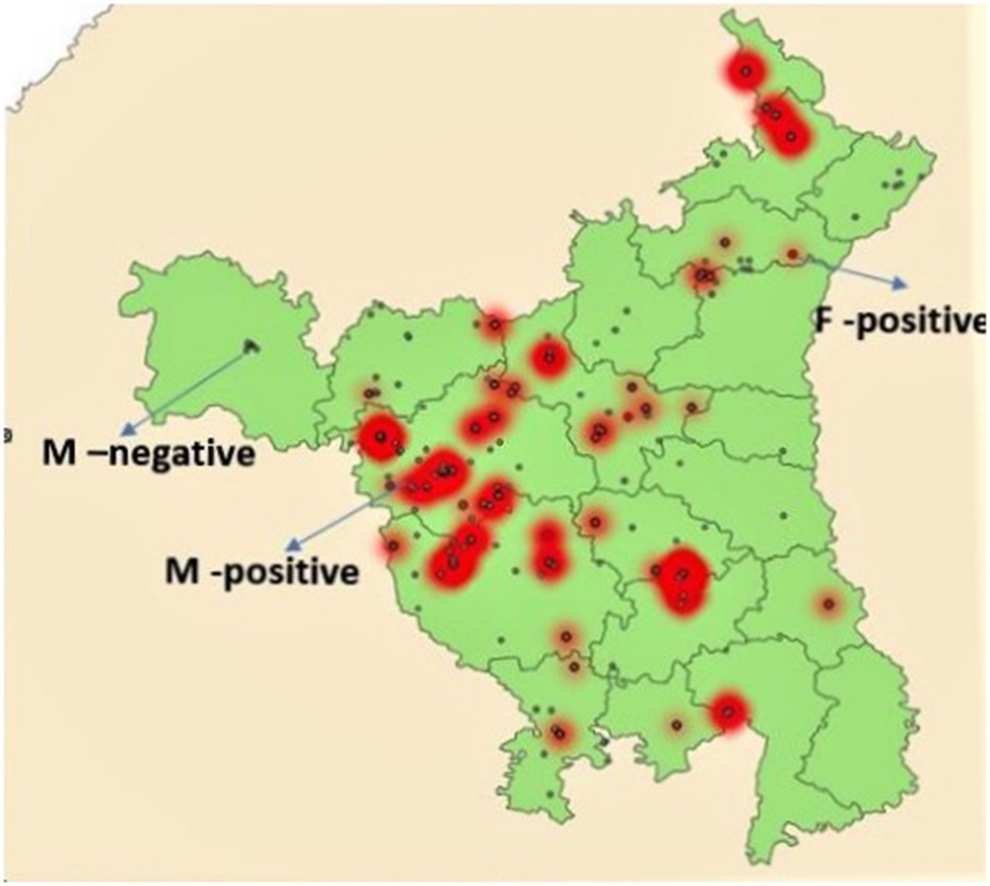
Map of Haryana showing locations of sample collection. Small red dots indicate F gene positive areas while red areas with gray dots indicate high positivity for M gene. Gray dots not in red areas indicate M gene negative areas.

Prevalence of NDV among different types of birds (backyard, commercial broilers, and commercial layers) is shown in Table 3. All prevalences in text are apparent prevalence, true prevalence is reported in Table 3. The prevalence was highest (196 of 1,657) among commercial broilers (11.8%; 95% CI: 10.4–13.5%), followed by commercial layers (77/782; 9.8%; 95% CI: 8.0–12.2%) and backyard poultry (119/1,562; 7.6%; 95% CI: 6.4–9.1%). The relative poultry population density maps for commercial and backyard poultry are given in Supplementary Figures 1, 2, respectively. When apparent prevalence was determined by type of housing system, it was highest for the intensive system (10.7%; 95% CI: 9.6–12.0%), with semi-intensive and extensive (backyard birds) at 8.25% (95% CI: 6.5–10.4) and 7.94% (95% CI: 6.1–10.3%), respectively. When analyzed according to the three zones, higher prevalence was observed in Zone 1 (13.2%; 95% CI: 11.5–15.1%) followed by Zone 3 (10.7%; 9.1–12.5%) and Zone 2 (5.6%; 4.5–6.9%). See Figure 1 for geographical information.

### Multivariable Logistic Regression Analysis

**Figure 2:**
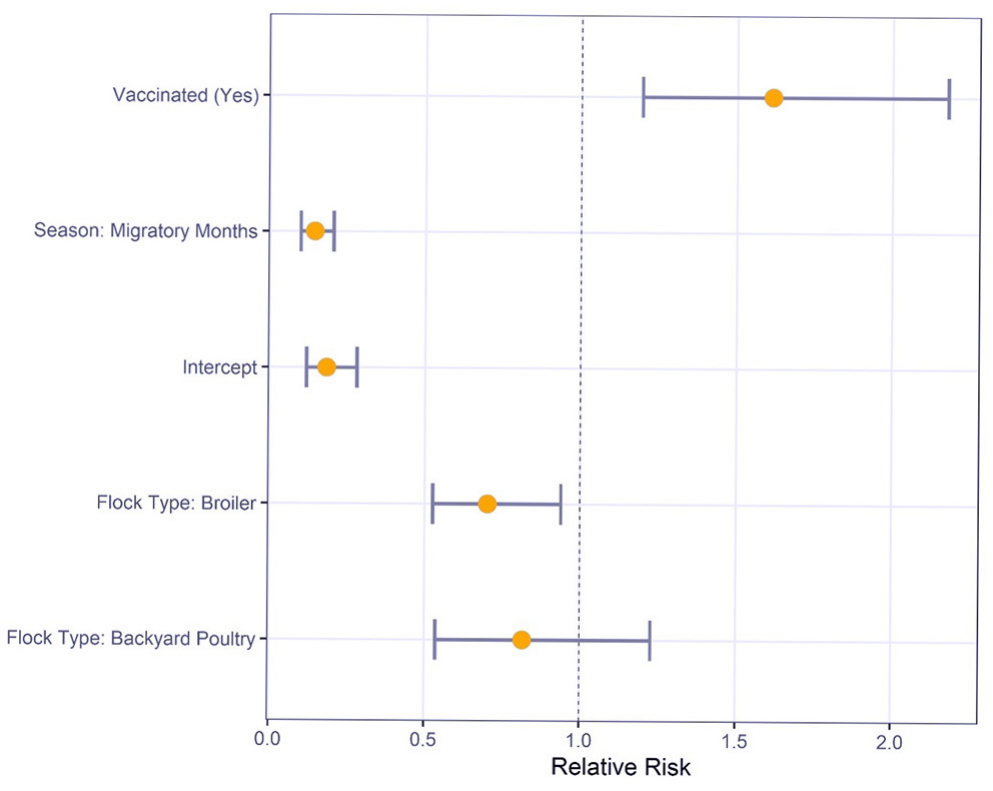
Relative Risk estimates and 95% confidence intervals for Newcastle disease virus M gene positive status by risk factors using multivariable logistic regression.

Multivariable logistic regression was used to understand the relative risk of various risk factors on M gene positive test results (Figure 2). For sampling conducted during the migratory months, there was a significantly reduced risk compared to sampling in non-migratory months (RR: 0.1, 95% CI: 0.1–0.2). Commercial broilers had a significantly lower risk of M gene positive test result than layers (RR: 0.7, 95% CI: 0.5–0.9). However, the difference between backyard poultry and layers was not statistically significant (RR: 0.8, 95% CI: 0.5–1.2).

### F Gene rRT-PCR

Of the 392 M gene positive samples, 35 (9.0%, 95% CI: 6.4–12.3%) were F gene positive (Table 4). Of the 196 samples from commercial broiler, 26 (13.3%; 95% CI: 9.0–19.0%) were F gene-positive, while in commercial layers 9 of 78 (11.5%; 95% CI: 5.7–21.3%) were positive. None of the 117 M gene-positive samples from backyard poultry were positive for the F gene. Areas with fusion gene positive samples are shown in Figure 1.

**Table 4:** Detection of mesogenic/velogenic strains of Newcastle disease virus according to bird type.

**F gene result**	**No. (%)**			
	**Commercial**	**Commercial**	**Backyard**	**Total**
	**broilers**	**layers**	**birds**	
Positive	26 (13.3)	9 (11.5)	0 (0.0)	35
Negative	170 (86.7)	69 (88.5)	117 (100)	356
Total	196	78	117	391

## Discussion

This study determined the circulation of NDV in commercial birds and backyard poultry in Haryana. The M gene rRT-PCR revealed that both commercial broilers and layers were NDV positive. Most of the M gene positive samples among non-vaccinated birds (107 of 146) belonged to backyard poultry (73.3%; 95% CI: 65.2–80.1%). It is interesting to note that all 35 F gene positive samples (8.9%; 95% CI: 6.4–12.3%) belonged to commercial broilers and layers, and none among backyard poultry. Vaccines based on lentogenic and mesogenic strains of NDV are used in breeder and commercial layer flocks while only lentogenic strains are used as vaccine in commercial broiler chicks in the state of Haryana. Most of the samples collected in this study were from commercial broilers chickens reared by small and marginal farmers. The farmers at the time of sampling could not provide specific input about the vaccine used. Therefore, we did not analyse the results with the vaccination program.

Backyard poultry is mostly reared by poor and marginal farmers in the state of Haryana. In this study, the questionnaire administered at the time of sampling indicated that no NDV vaccination was implemented among backyard poultry. The samples from backyard poultry that were M gene positive, but F gene negative may indicate circulation of avirulent NDV strains in backyard birds. This raises serious concerns regarding potential spill over of the circulating vaccine strains from commercial birds to backyard poultry leading to further virus evolution. The commercial farms are invariably situated at a distance of at least 3–4 kilometers from the area(s) where backyard poultry is kept. Further, the workers working in commercial farms are different from those maintaining the household backyard poultry. The finding that NDV is endemic in backyard poultry is similar to earlier work in other regions including West Africa (22).

Most of the previous studies undertaken in backyard poultry are based on detection of NDVs by molecular assay using F gene-specific primers or by determining antibody titers for NDV. Kouakou et al. (23) collected tracheal and cloacal swab samples from backyard and commercial poultry farms and live poultry markets in Ivory-Coast and tested 4,562 pooled samples for NDV using nested-PCR. Of the 4,562 pooled samples, 670 (14.7%) were found NDV positive. Ogali et al. (24) detected and characterized NDVs in rural backyard poultry farms in Kenya. Using partial F gene amplification assay, these authors reported that 2.7% (33/1,224) of samples from backyard poultry were positive for NDV. A serosurvey in Bushehr province in Iran found 40% of non-vaccinated chickens positive for NDV antibodies highlighting the need of molecular surveys in backyard poultry to confirm the circulating strains of NDV (25).

The circulation of lentogenic NDV among the backyard bird population in this study is in contrast with others (23, 26, 27) in which virulent NDVs were recorded in apparently healthy backyard poultry. The role of backyard poultry in the epidemiology of NDV in India is not fully understood. It is important to understand the genetic diversity of NDV strains circulating in backyard poultry in this region and their correlation with those in commercial poultry, if any (28). Such studies would help in designing long-term vaccination strategies. In the present study, sampling was done only once from the backyard units; however, regular monitoring of backyard poultry (a longitudinal study) may help better understand the types of NDV harbored by these birds over time. It is not only the virulent NDVs that cause economic losses to the farmers; even lentogenic strains can do so (29). For example, administration of 0, 4, 6, and 8 doses of LaSota vaccine (live vaccine) in broiler chicks resulted in decreased body weight gain and increased feed conversion ratio at all doses (29).

Most of the 35 F gene-positive samples in this study were from commercial poultry (21 broilers and 9 layers). The F gene positivity reflects the presence of mesogenic or velogenic strains. Low virulence lentogenic (F and LaSota) or mesogenic (Mukteswar) strains of NDV are used to vaccinate domestic poultry in India; the former is commonly used in commercial broilers while both types are used in commercial layers and breeders. Of these 35 F gene positive samples, 26 belonged to commercial broiler chickens. As per adopted practices in this region, no vaccination with mesogenic/velogenic strains is carried out in commercial broiler chickens. None of the matrix gene-positive samples from backyard poultry was positive by the fusion gene rRT-PCR. Future studies with monitoring of F gene positive apparently healthy flocks would be needed to ascertain whether this positivity translates into the disease or not. Recent studies have indicated the circulation of highly virulent velogenic NDV in apparently healthy backyard poultry with a high genetic gap from vaccinal strains (26). It has been reported that currently used NDV vaccines give better protection against the velogenic NDV isolated from 1930 to 1970s (Herts33/56, California 71) as compared to those which have been isolated in recent years (30).

The persistence of virulent NDV after intensive vaccination is a recurrent phenomenon in endemic countries of Asia, Africa, and Central America (6, 30–32), which is probably due to selection of virulent NDV in the face of immune pressure from vaccination (11). It also suggests that vaccination may stop active disease manifestation and protect the flock from an outbreak situation but may not stop shedding of the virus. As mentioned earlier, strains of low virulence are used as vaccines; however, experimental studies have suggested the evolution of virulent strains from low virulent NDV (33). Shengqing et al. (34) reported that avirulent NDV has the potential to become velogenic NDV after repeated passages in chickens. The replacement of Leu (avirulent) to Phe (virulent) amino acid in the F1 subunit makes F gene cleavage easier by host furin proteases, which gives a selective advantage to velogenic strains to grow faster. In such a scenario, avirulent strains may revert to virulent with time and with inadequate biosecurity measures; thus, the disease may spread within and between farms. In the present study, data analysis based on the age of the bird indicated that both M and F gene positivity was higher in birds below the age of 7 weeks. This higher prevalence at earlier age may be attributed to poor herd immunity (35). We did not study the effect of season on circulation of NDVs in commercial and backyard poultry. However, Gedara et al. (36) reported significantly higher occurrence of NDV in summer season as compared to winter season in backyard poultry.

In conclusion, this investigation found that lentogenic and mesogenic/velogenic NDV strains are circulating in commercial birds in Haryana while lentogenic strains are present in backyard birds predominantly. Detection of mesogenic/velogenic strains is not surprising taking into consideration the intensive system of rearing of commercial poultry in Haryana. NDV surveillance in non-outbreak situations could improve scientific understanding of the evolutionary patterns of NDV. A surveillance approach would also generate valuable information about the risk areas, distribution of the virus among various types of commercial and backyard poultry across various management systems, densities, and circulation patterns of virulent NDV in the region. Further studies should also explore whether commercial poultry flocks positive for velogenic NDV go on to develop clinical disease or not. Longitudinal studies are recommended to resolve this issue. A representative number of samples from this study are being subjected to whole genome sequencing to determine the presence of NDV genotypes and other viruses.

## Data Availability Statement

The original contributions presented in the study are included in the article/Supplementary Material, further inquiries can be directed to the corresponding author/s.

## Ethics Statement

The animal study was reviewed and approved by Institutional Animal Ethics Committee (LUVAS).

## Author Contributions

SG, VK, NJ, NM, SMo, SMa, and VJ: conceptualization. JR-B, CMH, SMo, VJ, and NJ: data curation. CMH, VJ, NJ, and SMa: formal analysis. SG, VK, NJ, and NM: funding acquisition. SMo, NM, VK, SG, NJ, SMa, VJ, and CMH: investigation methodology. SG and VK: project administration. SG, VK, NJ, PG, and NM: resources and supervision. CMH, JR-B, and SMo: software and validation. NB, DC, RS, VJ, CMH, NM, NJ, and SMa: sample collection and experiments. NJ, NM, SMa, SG, SMo, VK, JR-B, VJ, and CMH: visualization. VJ, SMa, NJ, NM, and SMo: writing original draft. VJ, DC, NB, RS, SMa, NM, CR, NS, SMo, JR-B, CMH, VK, PG, NJ, and SG: writing—review and editing. All authors contributed to the article and approved the submitted version.

## Funding

This project was funded by a United States Department of Defense, Defense Threat Reduction Agency, Biological Threat Reduction Program, Broad Agency Announcement grant HDTRA1-17-1-0045.

## Author Disclaimer

The opinions or assertions contained herein are the views of the authors and do not necessarily reflect the position or the policy of the sponsors and no official endorsement should be inferred.

## Conflict of Interest

The authors declare that the research was conducted in the absence of any commercial or financial relationships that could be construed as a potential conflict of interest.

## Publisher's Note

All claims expressed in this article are solely those of the authors and do not necessarily represent those of their affiliated organizations, or those of the publisher, the editors and the reviewers. Any product that may be evaluated in this article, or claim that may be made by its manufacturer, is not guaranteed or endorsed by the publisher.
