# Supplementary material for: Prevalence of Newcastle Disease Virus in Commercial and Backyard Poultry in Haryana, India
Source: Front Vet Sci. 2021 Nov 4;8:725232. doi: 10.3389/fvets.2021.725232 (PMC8600042; doi:10.3389/fvets.2021.725232)
Supplement: Supplementary file 1 [file Table_1.DOCX]

**SUPPLEMENTARY TABLE 1.** Districts in different zones of Haryana state

| Regions | Zone 1 | Zone 2 | Zone 3 |
| --- | --- | --- | --- |
| Districts | Sirsa, Fatehabad, Hisar, Jind, Bhiwani | Panchkula, Ambala, Kurukshetra, Yamunanagar, Kaithal | Sonipat, Jhajjar, Panipat, Karnal, Rohtak, Rewari, Mahendergarh, Nuh, Gurugram, Faridabad, Palwal |

**SUPPLEMENTARY TABLE 2.** Details of variables and information collected at the time of sampling

| **Category** | **Information** |
| --- | --- |
| Management | Housing type |
|  | Flock size |
|  | Age of birds |
| Location | Zone |
|  | Village, district, geo-coordinates |
|  | Owner details |
| Bird condition | Apparently healthy |
|  | Mild respiratory |
|  | Sick  Dead |
| Vaccination status | Vaccinated or not |
|  | Age at vaccination |
|  | Vaccine used/preferred |


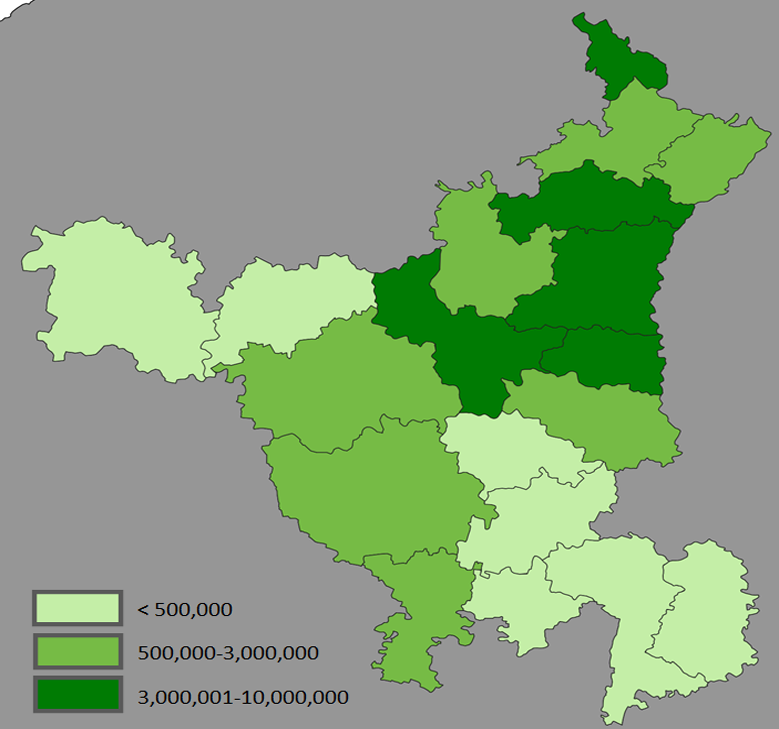


N

**SUPPLEMENTARY FIGURE 1**. Relative population density of commercial poultry birds in Haryana (37).


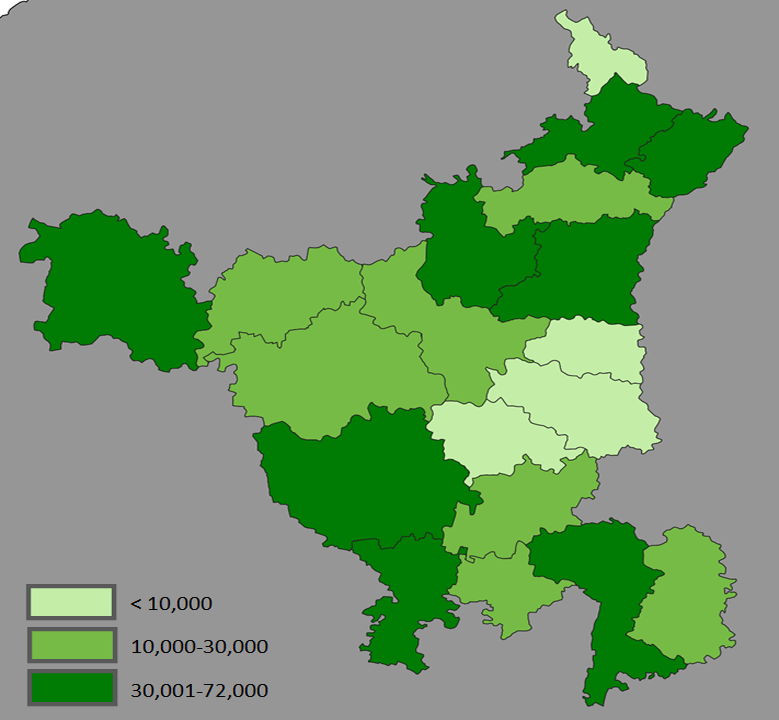


N

**SUPPLEMENTARY FIGURE 2**. Relative population density of backyard poultry birds in Haryana (37).
